# Supplementary material for: Cardiomyocyte-Specific Loss of Glutamyl-prolyl-tRNA Synthetase Leads to Disturbed Protein Homeostasis and Dilated Cardiomyopathy
Source: Cells. 2023 Dec 22;13(1):35. doi: 10.3390/cells13010035 (PMC10778562; doi:10.3390/cells13010035)
Supplement: Supplementary file 1 [file cells-13-00035-s001.zip › cells-2642887-supplementary.pdf]

Supplementary Information for

Cardiomyocyte-specific Loss of Glutamyl-prolyl-tRNA Synthetase Leads to Disturbed Protein Homeostasis and Dilated Cardiomyopathy

Jiangbin Wu, Jared Hollinger, Emily Bonanno, Feng Jiang, Peng Yao

Correspondence to Peng Yao

E-mail: peng_yao@urmc.rochester.edu

This document file includes:

Supplementary Figures 1 to 4

Supplementary Tables 1 to 2

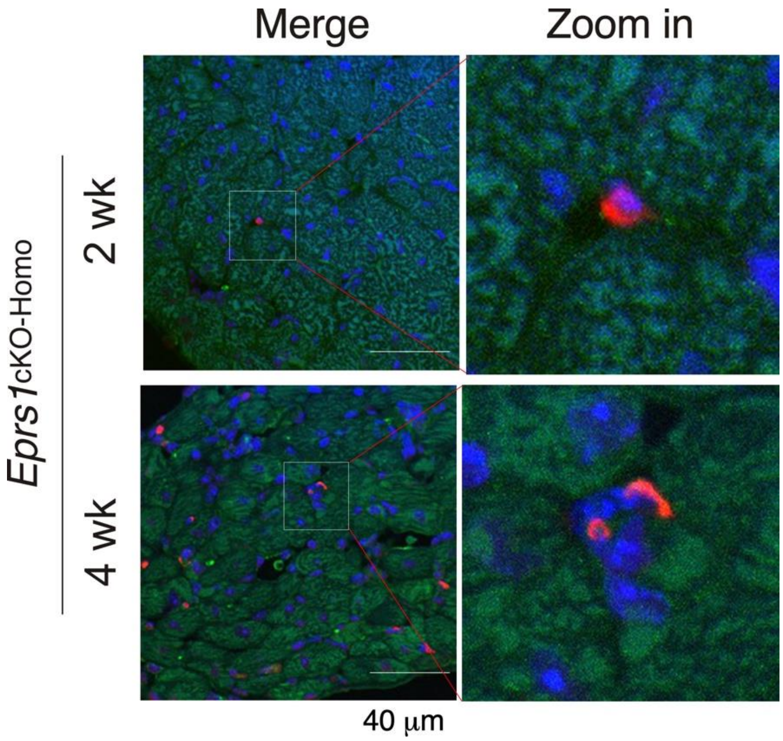

Figure S1. Zoom-in version of images containing positive TUNEL signals in Figure 2G.

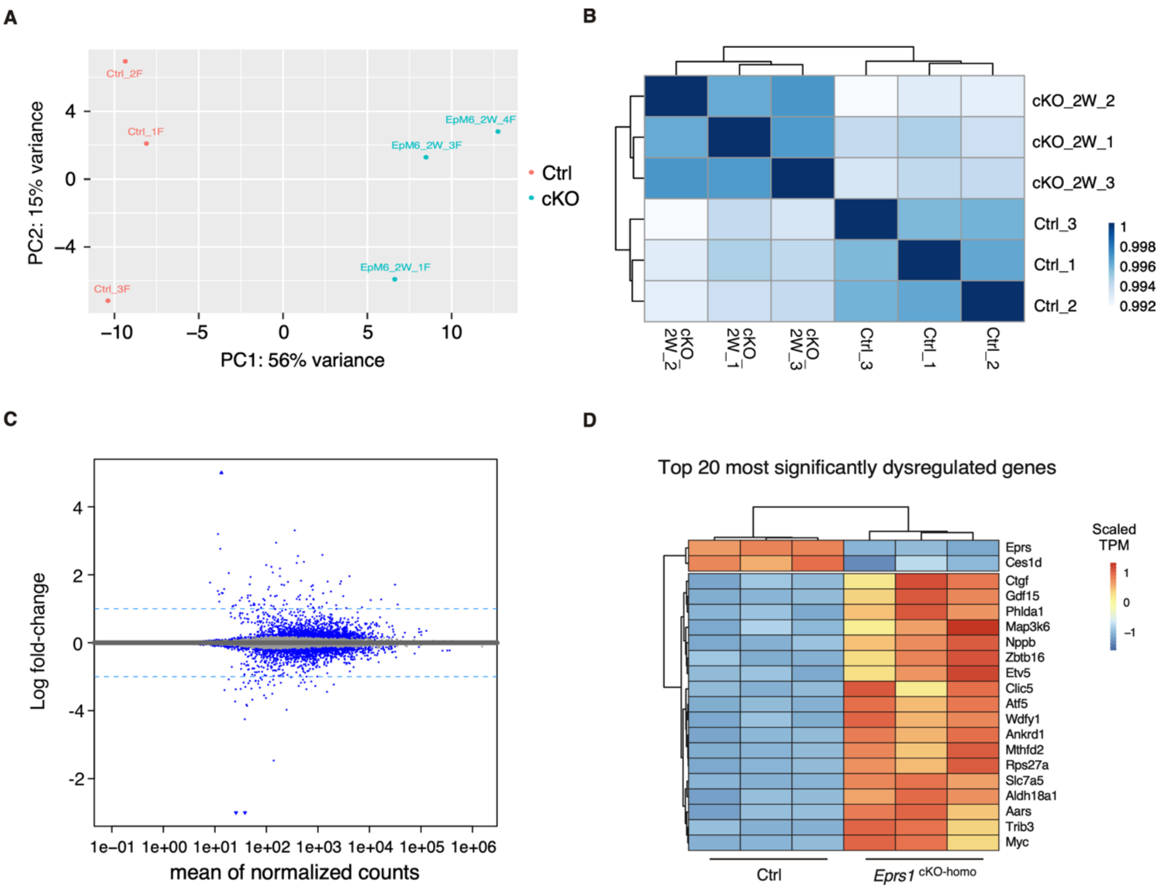

Figure S2. RNA-seq of Cardiomyocyte-specific *Eprs1* knockout and control mouse hearts.

(A) PCA analysis showing the distribution of the biological triplicates of RNA-seq for the control (Ctrl) and cardiomyocyte-specific EPRS cKO group.

(B) A heatmap showing the correlation among biological triplicates of RNA-seq. Pearson correlation was calculated and R^2 is shown in the heatmap.

(C) An M (log ratio) versus A (mean average) plot (MA-plot) showing the distribution of differentially expressed genes. Genes with significant *P* values ($P < 0.05$) are colored in blue.

(D) A heatmap showing the scaled TPM (transcript per million) of the top 20 most significantly dysregulated genes (sorted by *P* value) in all samples. Each row of the heatmap is scaled from -1 to 1.

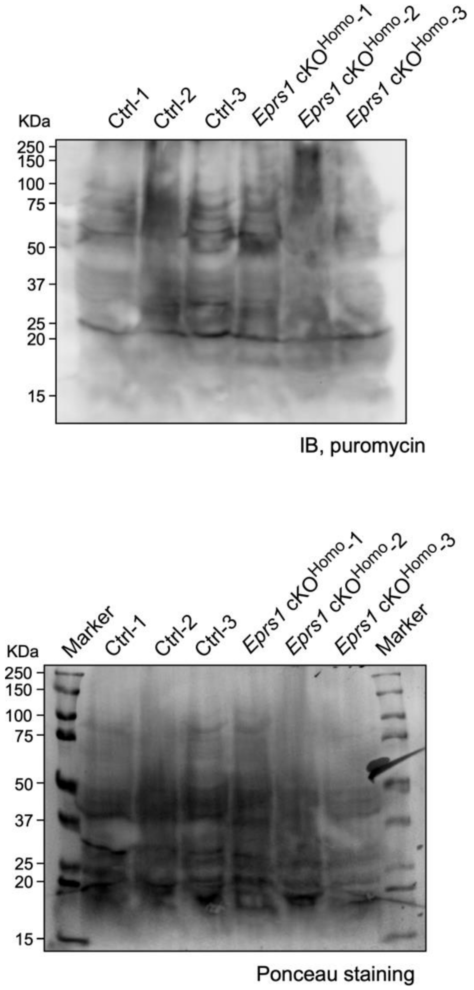

Figure S3. Puromycin incorporation followed by immunoblot confirms mild decrease in global protein synthesis in hearts upon *Eprs1* conditional knockout in CMs. (A) Western blot analysis of puromycin incorporation in control and *Eprs1* cKO hearts 2 weeks after tamoxifen injection. (B) Ponceau staining showing the loading for the total heart lysate protein samples.

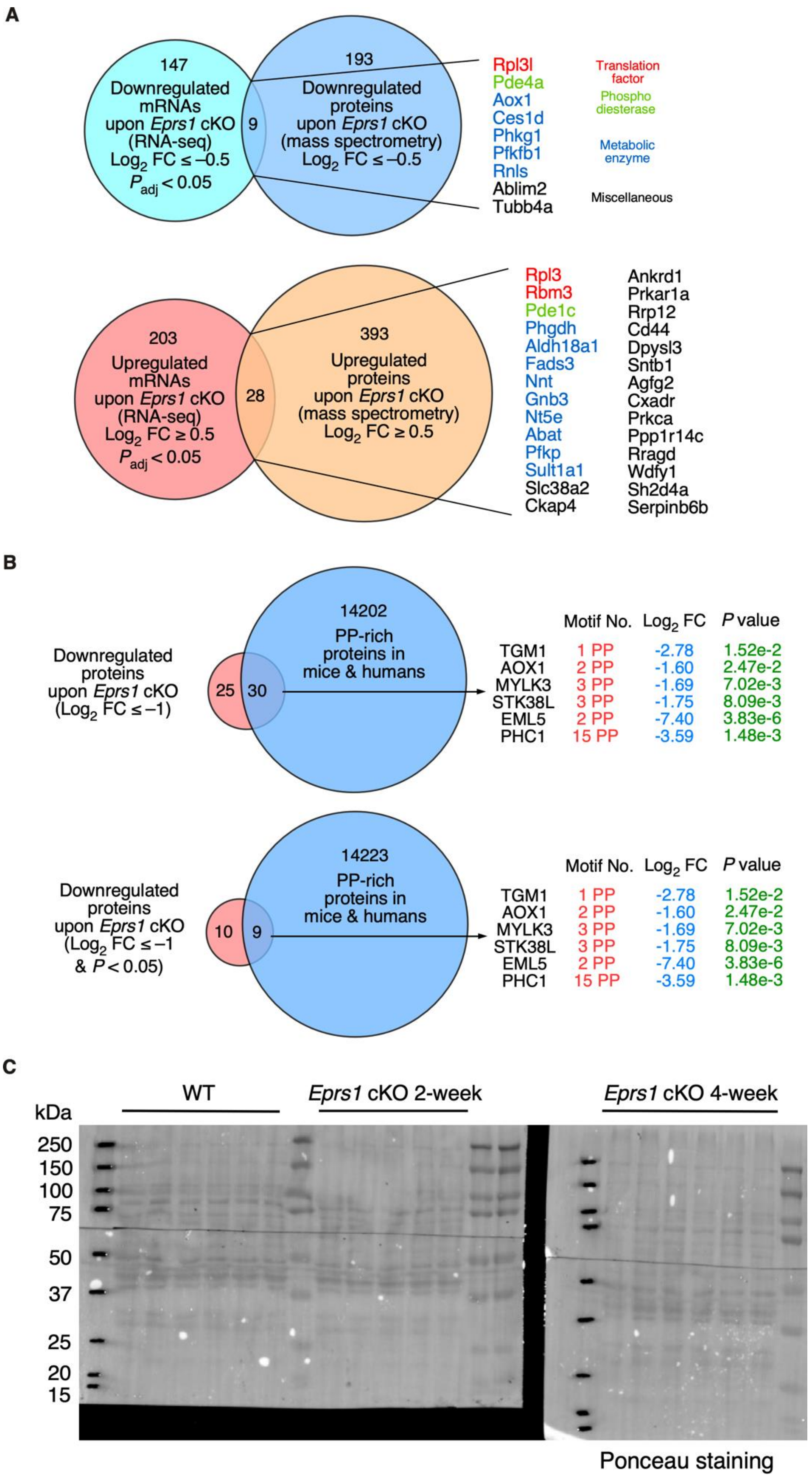
A
147
Downregulated mRNAs upon Eprs1 cKO (RNA-seq) Log2 FC ≤ –0.5 Padj < 0.05
9
193
Downregulated proteins upon Eprs1 cKO (mass spectrometry) Log2 FC ≤ –0.5
Rpl3l
Pde4a
Aox1
Ces1d
Phkg1
Pfkfb1
Rnls
Ablim2
Tubb4a
Translation factor
Phospho diesterase
Metabolic enzyme
Miscellaneous
203
Upregulated mRNAs upon Eprs1 cKO (RNA-seq) Log2 FC ≥ 0.5 Padj < 0.05
28
393
Upregulated proteins upon Eprs1 cKO (mass spectrometry) Log2 FC ≥ 0.5
Rpl3
Rbm3
Pde1c
Phgdh
Aldh18a1
Fads3
Nnt
Gnb3
Nt5e
Abat
Pfkp
Sult1a1
Slc38a2
Ckap4
Ankrd1
Prkar1a
Rrp12
Cd44
Dpysl3
Sntb1
Agfg2
Cxadr
Prkca
Ppp1r14c
Rragd
Wdfy1
Sh2d4a
Serpinb6b
B
Downregulated proteins upon Eprs1 cKO (Log2 FC ≤ –1)
25
30
14202
PP-rich proteins in mice & humans
Motif No. Log2 FC P value
TGM1 1 PP -2.78 1.52e-2
AOX1 2 PP -1.60 2.47e-2
MYLK3 3 PP -1.69 7.02e-3
STK38L 3 PP -1.75 8.09e-3
EML5 2 PP -7.40 3.83e-6
PHC1 15 PP -3.59 1.48e-3
Downregulated proteins upon Eprs1 cKO (Log2 FC ≤ –1 & P < 0.05)
10
9
14223
PP-rich proteins in mice & humans
Motif No. Log2 FC P value
TGM1 1 PP -2.78 1.52e-2
AOX1 2 PP -1.60 2.47e-2
MYLK3 3 PP -1.69 7.02e-3
STK38L 3 PP -1.75 8.09e-3
EML5 2 PP -7.40 3.83e-6
PHC1 15 PP -3.59 1.48e-3
C
WT
Eprs1 cKO 2-week
Eprs1 cKO 4-week
kDa
250
150
100
75
50
37
25
20
15
Ponceau staining

Figure S4. (A) Venn diagrams showing the overlapped genes between dysregulated mRNAs and proteins based on RNA-seq and mass spectrometry analysis in hearts samples from control and cKO hearts in mice at 2 weeks post-tamoxifen injections. **(B)** Reanalysis of Venn diagram showing downregulated proteins containing Pro-Pro dipeptidyl motifs in *Eprs1* cKO hearts using two more stringent cut-offs. **(C)** Representative ponceau staining images to indicate equal loading of the total heart lysate proteins for western blot of TGM1.

Table S1. Differentially expressed genes in *Eprs1* cKO hearts compared to control hearts at the early stage identified by RNA-Seq. Gene expression changes of ribosome protein-coding mRNAs are highlighted. Gene Ontology analysis data is shown.

Table S2. Differentially expressed proteins in *Eprs1* cKO hearts compared to control hearts at the early stage were identified by quantitative mass spectrometry analysis. Gene Ontology analysis data is shown. A pro-Pro dipeptidyl motif-bearing protein list is included.
